# Influence of Age of Onset on Huntington’s Disease Phenotype

**DOI:** 10.5334/tohm.536

**Published:** 2020-07-09

**Authors:** Lauren Kwa, Danielle Larson, Chen Yeh, Danny Bega

**Affiliations:** 1 Northwestern University Feinberg School of Medicine, US; 2 Northwestern University Feinberg School of Medicine, Department of Neurology, US; 3 Department of Preventive Medicine-Biostatistics, Northwestern University Feinberg School of Medicine, US

**Keywords:** Huntington Disease (HD), Enroll-HD, phenotype, UHDRS, TFC

## Abstract

**Background:**

Older patients with Huntington’s disease (HD) are often thought to have a slower progressing disease course with less behavioral symptoms than younger patients. However, phenotypic differences based on age of onset have not been well characterized in a large HD population. This study will determine the difference in manifestations and disease progression between patients with young, typical, and late onset adult HD at different stages of disease.

**Methods:**

Data obtained from Enroll-HD. Adults with manifest HD were included. Age groups were defined as young onset (YO: 20-29 years), typical onset (TO: 30–59 years), and late onset (LO: 60+ years). Subjects were categorized by TFC score, from Stage I (least severe) to Stage V (most severe). Motor, cognitive, and behavioral symptoms were analyzed. Descriptive statistics and Bonferroni p-value correction for pairwise comparison were calculated.

**Results:**

7,311 manifest HD participants were included (612 YO, 5,776 TO, and 923 LO). The average decline in TFC score from baseline to second visit (1.5–2.5 years) was significantly faster for YO (–1.75 points) compared to TO (–1.23 points, p = 0.0105) or LO (–0.97 points, p = 0.0017). Motor deficits were worse for LO participants at early stages of HD, and worse for YO participants at advanced stages. YO and TO participants had greater burden of behavioral symptoms at early stages of disease compared to LO.

**Discussion:**

YO is predictive of a faster functional decline for adults with HD when compared to those with TO and LO. Motor and behavioral manifestations differ based on age of onset.

**Highlights:**

This study compares HD manifestations while controlling for disease severity, detailing robust phenotypic differences by age of onset alone. These findings have implications for the clinical management of HD symptoms and have the possibility to improve prognostic and treatment precision.

## Introduction

Huntington’s Disease (HD) is an inherited neurodegenerative disorder that is characterized by progressive motor, cognitive, and behavioral decline. It is inherited in an autosomal dominant pattern, with the number of CAG repeats in the HTT gene being the strongest determinant of age of onset [1]. Average age of onset is about 40 years old, with a range from childhood to the eighth decade [2,3]. It has been suggested that older onset patients are more likely to present with motor manifestations, and younger patients with psychiatric/behavioral manifestations [4]. However, phenotypic differences by age of onset have not been well-characterized in the HD literature, likely due to sample size limitations of most studies. Furthermore, whether phenotypic differences persist or vary by disease stage has not been previously described. There remains a lack of clarity on the relationship between age of onset and HD progression. In a 1995 study, Feigin et al. reported no significant connection between rate of functional decline and age of onset [5]. A more recent publication described a significant association between younger age of onset and faster decline in UHDRS motor score and Independence Scale, but did not find an association with Total Functional Capacity (TFC) [6]. Using the large database of HD patients from Enroll-HD, we characterized HD phenotype and functional decline by age group across the spectrum of disease severity. Knowledge of the phenotypic profile and rate of progression by age of onset will inform standard of care recommendations and aim to improve the quality of care for HD patients.

## Methods

This study is a cross-sectional analysis of prospectively collected data from manifest HD subjects in the Enroll-HD database. Enroll-HD began in 2012 as part of an observational, multi-national, multi-center study and contains longitudinal clinical information on over 15,000 participants as of December 2018. This dataset includes 3,802 participants from the European REGISTRY study that preceded Enroll-HD [7]. Study participants are recruited from specialty clinics and enrolled from 125 sites located in 13 countries across four continents. Each study site is evaluated by an IRB or equivalent ethics committee. Enroll-HD collects deidentified data from participants at annual study visits and utilizes a risk-based monitoring strategy to maintain data quality [8]. The data for this study was from the dataset cutoff made available in December 2018.

Participants with manifest/motor-manifest HD at enrollment were included. Participants younger than 20 years of age at symptom onset were excluded to remove those with juvenile-onset Huntington’s disease, which differs significantly in phenotype from adult-onset HD and has already been well-characterized in the literature as a distinct clinical presentation [9,10]. Age groups were defined as follows: young onset (20–29 years), typical onset (30–59 years), and late onset (60+ years). Typical and late onset age group cutoffs have been used and validated by previous studies [11,12,13,14,15]. Age of onset was determined by Enroll-HD rater’s estimate of symptom onset. Subjects in each group were categorized into TFC total score bins, from Stage I (least severe) to Stage V (most severe). TFC Total Score 11–13 is characterized as Stage I, 7–10 is Stage II, 3–6 is Stage III, 1–2 is Stage IV, and 0 is Stage V. For the purposes of analysis, TFC scores were grouped into 3 previously validated categories: early (stage I and II), moderate (stage III), and advanced (stages IV and V) [16,17]. These TFC score stages are utilized in clinical practice and in contemporary research studies [13,18,19].

Demographic data and motor, cognitive, and behavioral measures were analyzed. Motor variables included the Unified Huntington’s Disease Rating Scale (UHDRS) motor score and Timed “Up and Go” test. Cognitive variables included Symbol Digit Modality Test, Verbal Fluency Test (Category), Verbal Fluency Test (Letter), Stroop Color Naming Test, Stroop Word Reading Test, and Stroop Interference Test. For the above measures, percentage correct was calculated by dividing total correct answers by total answers. Percentage correct was used for analyses. Mini Mental State Examination (MMSE) score was also included as a measure of cognitive function.

Behavioral measures included quantification of drug and alcohol abuse; Hospital Anxiety and Depression Scale – Snaith Irritability Scale (HADS-SIS) anxiety, depression, irritability, outward irritability, and inward irritability subscores, total number of suicide attempts (from Columbia-Suicide Rating Scale baseline and follow-up); and Problem Behaviours Assessment – Short (PBA-s) Depression, Irritability aggression, Psychosis, Apathy, Executive function, Depressed mood frequency, Suicidal ideation frequency, Anxiety frequency, Irritability frequency, Angry or aggressive behavior frequency, Lack of initiative (apathy) frequency, Perseverative thinking or behavior frequency, Obsessive-Compulsive Behaviors frequency, Delusions frequency, Hallucinations frequency, Disoriented Behavior frequency. For all PBA-s frequency variables, responses were converted to and analyzed as binary variables: not present (coded as *0-absent*) vs. present (includes *1-slight, questionable, 2-mild, 3-moderate*, and *4-severe*).

### Data Analysis

Descriptive statistics were calculated for all variables of interest. Categorical variables were summarized with counts and percentages, and continuous variables with means and standard deviations or median and interquartile range, as appropriate. The drug and alcohol abuse variables were binary variables (yes/no). TFC score at baseline was utilized to sort participants into early, moderate, and advanced disease stage. Chi-square test, Fisher’s exact test, and Kruskal-Wallis test, as appropriate, were used to determine p-values. P-values less than 0.05 were considered significant for overall comparison. As we aimed to identify any factors potentially associated with age of onset, we did not consider p-value correction on potential factors. However, Bonferroni p-value correction was used to do pairwise comparison for post-hoc analysis. Bonferroni corrected p-values less than 0.01667 were considered significant when we compared 3 combinations of age of onset groups. In this context, we viewed control over type II error more important than control over type I error. In addition, the analyses were deemed exploratory overall and the primary focus was not to generate a precise, predictive model.

Sensitivity analysis was conducted to determine the average change in TFC score between age of onset groups, excluding participants whose first visit was outside 6 months to 1.5 years after baseline visit, participants whose second visit was outside 1.5 year to 2.5 years after baseline, and participants whose third visit was outside 2.5 years to 3.5 years after baseline. Follow-up visits for Enroll-HD are to be conducted annually, and these exclusions remove variation from that timeline. Additionally, only participants who had TFC scores for their baseline, first visit, second visit, and third visit were included. ANOVA was used to compare mean TFC score change from baseline between age of onset groups. The same parameters were used for a sensitivity analysis of average change in UHDRS Functional Assessment Independence Scale.

## Results

7,311 manifest HD subjects were included in the analysis of which 612 were young onset, 5,776 typical onset, and 923 late onset. There were 15,301 total participants in the Enroll-HD database, of which 8,043 had manifest HD. 176 participants were excluded due to age <20 and 556 were excluded due to missing age of onset value. Participants were 51.5% female, 94.3% white/Caucasian, and average CAG repeats in the HTT gene was 43.8. The average age of clinical HD diagnosis in our adult study population was 49.2 years (Table 1).

**Table 1:** Demographic Characteristics.

	Age of Onset Groups						ALL	

	Young onset (20–29 years)		Typical onset (30–59 years)		Late onset (60+ years)			

	N	%	N	%	N	%	N	%

TFC stage								
Stage I (11–13)	191	31.31	1864	32.30	278	30.18	2333	31.95
Stage II (7–10)	198	32.46	2025	35.09	347	37.68	2570	35.20
Stage III (3–6)	139	22.79	1265	21.92	215	23.34	1619	22.17
Stage IV (1–2)	54	8.85	445	7.71	67	7.27	566	7.75
Stage V (0)	28	4.59	172	2.98	14	1.52	214	2.93
TFC stage								
Early (Stage I & II)	389	63.77	3889	67.39	625	67.86	4903	67.15
Moderate (Stage III)	139	22.79	1265	21.92	215	23.34	1619	22.17
Advanced (Stage IV & V)	82	13.44	617	10.69	81	8.79	780	10.68
Sex								
Female	319	52.12	2998	51.90	445	48.21	3762	51.46
Male	293	47.88	2778	48.10	478	51.79	3549	48.54
Race								
Other	41	6.70	335	5.80	39	4.23	415	5.68
White/Caucasian	571	93.30	5441	94.20	884	95.77	6896	94.32
Has Mother Affected	279	46.66	2660	47.75	365	45.23	3304	47.36
Has Father Affected	308	51.85	2602	47.12	278	34.71	3188	46.09
Has Family History	526	85.95	4866	84.25	788	85.37	6180	84.53
Marital Status								
Married/Partnership	199	32.52	3782	65.56	691	74.95	4672	63.97
Single/Divorced/Widowed/Legally Separated	413	67.48	1987	34.44	231	25.05	2631	36.03
ISCED Education Level								
Less than or equal to 12th grade	374	61.41	3217	55.96	541	58.87	4132	56.78
Higher than 12th grade	235	38.59	2532	44.04	378	41.13	3145	43.22
Employment Status								
Employed	129	21.11	1302	22.59	79	8.60	1510	20.70
Not Employed	482	78.89	4461	77.41	840	91.40	5783	79.30
Rater’s Judgement of Initial Major Symptom								
Motor	269	44.39	2966	51.51	631	68.36	3866	53.05
Cognitive	46	7.59	489	8.49	42	4.55	577	7.92
Psychiatric	165	27.23	1197	20.79	104	11.27	1466	20.12
Oculomotor/Other/Mixed	126	20.79	1106	19.21	146	15.82	1378	18.91
	Mean	SD	Mean	SD	Mean	SD	Mean	SD
BMI	24.32	5.43	25.03	5.03	25.02	4.31	24.97	4.99
Age of clinical HD diagnosis	29.83	6.25	48.27	8.72	67.92	5.57	49.22	11.99
Larger research CAG allele determined from DNA	49.81	4.67	43.59	2.49	40.75	1.19	43.75	3.34

Missing values were encountered in 9 (0.12%) for TFC score, 335 (4.58%) for mother affected, 394 (5.39%) for father affected, 8 (0.11%) for marital status, 34 (0.47%) for education level, 18 (0.25%) for employment status, 24 (0.33%) for rater’s judgement of initial major symptom, 235 (3.21%) in BMI, and 140 (1.91%) in age of clinical HD diagnosis.

### Motor Variables

At early (TFC I-II) and advanced (TFC IV-V) stages of disease, motor function varied based on age of onset. At early stages of disease, the late age of onset group had worse motor function compared to the young and typical age of onset groups, with significantly worse UHDRS Motor scores in late onset (median [Q1–Q3] = 30.00 [20.00–39.00]) compared to young onset (25.00 [15.00–38.00], p-value = 0.00007) and typical onset participants (27.00 [18.00–38.00], p = 0.0003).

At advanced stages of disease, all the age groups had significantly different motor function. The young onset group had worse UHDRS motor scores (83.00 [70.00–91.50]) compared to the typical onset (75.00 [63.00–87.00], p = 0.003) and late onset participants (65.00 [53.00–77.00], p < 0.001). The typical onset group also had significantly worse motor scores compared to the late onset group (p < 0.001) (Table 2).

**Table 2:** Motor and Cognitive Variables by Age of Onset Groups and TFC Stages.

	TFC Score Category	Young		Typical			Late	P-value

		N	Median (Q1–Q3) or N (%)	N	Median (Q1–Q3) or N (%)	N	Median (Q1–Q3) or N (%)	

**Motor Symptoms**								
UHDRS Motor score (TMS)	Early	387	25.00 (15.00, 38.00)	3871	27.00 (18.00, 38.00)	619	30.00 (20.00, 39.00)	* <0.01
	Moderate	139	56.00 (39.00, 67.00)	1248	51.00 (38.50, 63.00)	214	51.00 (39.00, 60.00)	0.13
	Advanced	80	83.00 (70.00, 91.50)	609	75.00 (63.00, 87.00)	79	65.00 (53.00, 77.00)	* <0.01
Timed “Up and Go” Total time	Early	190	8.75 (7.00, 11.00)	1661	9.00 (7.40, 11.00)	252	10.00 (8.80, 13.00)	* <0.01
	Moderate	45	11.00 (9.00, 15.00)	462	12.00 (9.50, 16.00)	63	14.00 (11.00, 20.00)	* 0.02
	Advanced	10	14.00 (12.00, 21.00)	72	15.50 (10.00, 23.00)	12	17.00 (15.00, 26.50)	0.47
**Cognitive Symptoms**								
Symbol Digit Modality Test Total correct %	Early	384	100.00 (93.75, 100.00)	3819	100.00 (95.00, 100.00)	609	100.00 (93.33, 100.00)	* 0.02
	Moderate	118	100.00 (86.67, 100.00)	1104	96.23 (86.36, 100.00)	187	95.00 (75.00, 100.00)	0.13
	Advanced	52	0.00 (0.00, 87.78)	398	0.00 (0.00, 94.74)	45	50.00 (0.00, 100.00)	0.16
Verbal Fluency Test Total correct % Category	Early	384	100.00 (93.22, 100.00)	3847	100.00 (90.91, 100.00)	622	100.00 (90.91, 100.00)	* 0.01
	Moderate	134	100.00 (87.50, 100.00)	1227	100.00 (85.71, 100.00)	210	100.00 (83.33, 100.00)	0.41
	Advanced	65	85.71 (0.00, 100.00)	491	85.71 (40.00, 100.00)	67	100.00 (75.00, 100.00)	* 0.03
Verbal Fluency Test Total correct % Letter	Early	300	95.45 (90.12, 100.00)	3037	95.00 (88.89, 100.00)	456	94.12 (87.50, 100.00)	0.18
	Moderate	92	94.28 (85.96, 100.00)	854	92.98 (82.61, 100.00)	155	90.00 (76.00, 100.00)	* <0.01
	Advanced	41	80.95 (0.00, 100.00)	277	76.47 (50.00, 100.00)	41	85.71 (61.54, 100.00)	0.44
Stroop Color Naming Test Total correct %	Early	381	100.00 (100.00, 100.00)	3833	100.00 (100.00, 100.00)	614	100.00 (100.00, 100.00)	0.84
	Moderate	133	100.00 (98.63, 100.00)	1206	100.00 (98.08, 100.00)	202	100.00 (97.50, 100.00)	0.78
	Advanced	64	98.49 (0.00, 100.00)	468	97.26 (0.00, 100.00)	60	98.53 (64.38, 100.00)	0.82
Stroop Word Reading test Total correct %	Early	381	100.00 (100.00, 100.00)	3831	100.00 (100.00, 100.00)	614	100.00 (100.00, 100.00)	0.18
	Moderate	129	100.00 (100.00, 100.00)	1187	100.00 (100.00, 100.00)	200	100.00 (100.00, 100.00)	0.65
	Advanced	63	96.67 (0.00, 100.00)	457	100.00 (0.00, 100.00)	60	100.00 (0.00, 100.00)	0.19
Stroop Interference Test Total correct %	Early	356	100.00 (96.08, 100.00)	3511	100.00 (96.15, 100.00)	548	100.00 (95.00, 100.00)	0.24
	Moderate	106	100.00 (88.89, 100.00)	956	100.00 (87.50, 100.00)	155	94.74 (76.92, 100.00)	* 0.02
	Advanced	38	95.00 (36.84, 100.00)	281	88.24 (16.67, 100.00)	33	84.62 (30.00, 100.00)	0.53
Mini Mental State Examination (MMSE) score	Early	270	26.00 (24.00, 29.00)	2562	27.00 (25.00, 29.00)	393	27.00 (25.00, 28.00)	0.13
	Moderate	80	24.00 (20.00, 26.50)	725	23.00 (20.00, 26.00)	128	23.00 (19.00, 25.00)	0.08
	Advanced	34	17.00 (11.00, 22.00)	252	17.00 (13.00, 21.00)	37	18.00 (15.00, 22.00)	0.68

* Significant at level p < 0.05.

### Cognitive Variables

The cognitive variables analyzed yielded no clinically significant trends when compared between groups. At early stages of HD, some intergroup differences were seen for Symbol Digit Modality Test (p = 0.02) and Verbal Fluency Test Category (p = 0.01). For instance, the young age of onset group scored slightly better (median [Q1–Q3] = 100.00 [93.22–100.00]) compared to typical (100.00 [90.91–100.00], p = 0.003) and late onset group (100.00 [90.91–100.00], p = 0.013) on the Verbal Fluency Test Category. At moderate stages of disease, late onset participants had significantly worse scores compared to typical onset participants compared on the Verbal Fluency Test Letter (90.00 [76.00–100.00] vs. 92.98 [82.61–100.00], p = 0.003) and Stroop Interference Test (94.74 [76.92–100.00] vs. 100.00 [87.50, 100.00], p = 0.011) (Table 2).

### Behavioral Variables

At early stages of disease, the young onset group tended to have significantly worse behavioral symptoms, including drug and alcohol abuse, anxiety, depression, irritability, aggression, apathy, lack of initiative, obsessive-compulsive behaviors, and delusions when compared to the late onset group (Table 3). At moderate stages of disease, the late onset group scored significantly worse on HADS-SIS depression and HADS-SIS inward irritability when compared to the young onset group. Young onset participants were more likely to abuse drugs compared to both the typical and late onset groups at early, moderate, and advanced stages of disease (all p < 0.01) (Appendix A). At advanced stages of disease, young onset participants were more likely to have delusions (29.27% vs. 16.02%, p < 0.009) and hallucinations (20.73% vs. 7.41%, p = 0.015) when compared to the late onset group (Table 3).

**Table 3:** Behavioral Variables by Age of Onset Groups and TFC Stages.

	TFC Score Category	Young		Typical		Late		P-value

		N	Median (Q1–Q3) or N (%)	N	Median (Q1–Q3) or N (%)	N	Median (Q1–Q3) or N (%)	

**Behavioral Symptoms**								
Has the participant ever abused drugs? Yes	Early	389	77 (19.79)	3883	385 (9.92)	622	23 (3.70)	* <0.01
	Moderate	139	27 (19.42)	1262	84 (6.66)	214	4 (1.87)	* <0.01
	Advanced	80	10 (12.50)	613	23 (3.75)	81	0 (0.00)	* <0.01
Has the participant had alcohol problems in the past? Yes	Early	389	50 (12.85)	3881	358 (9.22)	622	28 (4.50)	* <0.01
	Moderate	139	17 (12.23)	1261	146 (11.58)	214	15 (7.01)	0.13
	Advanced	81	8 (9.88)	612	64 (10.46)	81	5 (6.17)	0.55
HADS-SIS anxiety subscore	Early	264	6.00 (3.50, 9.00)	2417	6.00 (3.00, 9.00)	365	4.00 (2.00, 7.00)	* <0.01
	Moderate	62	4.00 (2.00, 8.00)	604	5.00 (3.00, 9.00)	100	5.00 (3.00, 8.00)	0.28
	Advanced	24	4.00 (2.50, 8.50)	172	5.00 (3.00, 9.00)	25	4.00 (2.00, 6.00)	0.40
HADS-SIS depression subscore	Early	264	5.00 (3.00, 8.00)	2416	5.00 (3.00, 8.00)	365	4.00 (2.00, 7.00)	* <0.01
	Moderate	62	5.00 (3.00, 8.00)	602	7.00 (4.00, 10.00)	99	8.00 (4.00, 11.00)	* <0.01
	Advanced	24	7.00 (3.00, 11.00)	176	8.00 (5.00, 12.00)	25	7.00 (6.00, 9.00)	0.39
HADS-SIS irritability subscore	Early	263	6.00 (3.00, 10.00)	2413	6.00 (2.00, 9.00)	365	5.00 (2.00, 7.00)	* <0.01
	Moderate	62	5.00 (2.00, 8.00)	601	5.00 (2.00, 9.00)	100	5.00 (3.00, 8.00)	0.52
	Advanced	25	4.00 (2.00, 8.00)	174	5.00 (2.00, 8.00)	25	4.00 (2.00, 7.00)	0.88
HADS-SIS outward irritability subscore	Early	263	4.00 (2.00, 7.00)	2426	3.00 (1.00, 6.00)	368	3.00 (1.00, 5.00)	* <0.01
	Moderate	62	3.00 (1.00, 6.00)	604	3.00 (1.00, 6.00)	101	3.00 (1.00, 5.00)	0.23
	Advanced	25	3.00 (1.00, 5.00)	175	3.00 (1.00, 6.00)	25	2.00 (1.00, 5.00)	0.65
HADS-SIS inward irritability subscore	Early	264	2.00 (0.00, 4.00)	2421	2.00 (0.00, 4.00)	365	2.00 (0.00, 3.00)	* <0.01
	Moderate	62	1.00 (0.00, 3.00)	603	2.00 (0.00, 4.00)	100	2.50 (1.00, 4.00)	* 0.01
	Advanced	25	1.00 (0.00, 3.00)	174	1.00 (0.00, 3.00)	25	1.00 (0.00, 3.00)	0.86
Total # of suicide attempts (C-SSRS BL and FUP)	Early	27	1.00 (1.00, 2.00)	142	1.00 (1.00, 2.00)	13	1.00 (1.00, 1.00)	0.55
	Moderate	19	1.00 (1.00, 2.00)	75	1.00 (1.00, 2.00)	7	1.00 (1.00, 2.00)	0.99
	Advanced	5	2.00 (1.00, 2.00)	32	1.00 (1.00, 2.50)	3	1.00 (1.00, 3.00)	0.75
PBA-s Depression	Early	388	4.00 (0.00, 9.50)	3883	4.00 (0.00, 8.00)	624	2.00 (0.00, 6.00)	* <0.01
	Moderate	138	2.50 (0.00, 7.00)	1257	3.00 (0.00, 8.00)	215	4.00 (0.00, 8.00)	0.50
	Advanced	71	2.00 (0.00, 8.00)	545	2.00 (0.00, 8.00)	78	2.50 (0.00, 8.00)	0.88
PBA-s Irritability aggression	Early	386	2.00 (0.00, 6.00)	3885	1.00 (0.00, 4.00)	624	0.50 (0.00, 4.00)	* <0.01
	Moderate	138	1.00 (0.00, 6.00)	1261	1.00 (0.00, 6.00)	215	1.00 (0.00, 4.00)	0.13
	Advanced	80	1.00 (0.00, 7.00)	599	2.00 (0.00, 8.00)	79	1.00 (0.00, 4.00)	0.32
PBA-s Psychosis	Early	386	0.00 (0.00, 0.00)	3878	0.00 (0.00, 0.00)	625	0.00 (0.00, 0.00)	0.17
	Moderate	137	0.00 (0.00, 0.00)	1257	0.00 (0.00, 0.00)	215	0.00 (0.00, 0.00)	0.06
	Advanced	69	0.00 (0.00, 0.00)	547	0.00 (0.00, 0.00)	77	0.00 (0.00, 0.00)	0.22
PBA-s Apathy	Early	387	1.00 (0.00, 6.00)	3884	0.00 (0.00, 4.00)	624	0.00 (0.00, 4.00)	* <0.01
	Moderate	138	3.00 (0.00, 8.00)	1258	4.00 (0.00, 9.00)	215	6.00 (0.00, 9.00)	0.10
	Advanced	71	8.00 (0.00, 12.00)	555	8.00 (0.00, 12.00)	77	8.00 (1.00, 12.00)	0.97
PBA-s Executive function	Early	386	0.00 (0.00, 4.00)	3874	0.00 (0.00, 4.00)	623	0.00 (0.00, 4.00)	* <0.01
	Moderate	137	4.00 (0.00, 9.00)	1256	3.00 (0.00, 9.00)	215	2.00 (0.00, 6.00)	0.16
	Advanced	71	6.00 (0.00, 12.00)	541	6.00 (0.00, 10.00)	76	4.00 (0.00, 9.00)	0.34
PBA-s Depressed mood frequency Present vs. Not Present	Early	389	224 (57.58)	3889	2064 (53.07)	625	267 (42.72)	* <0.01
	Moderate	139	60 (43.17)	1265	623 (49.25)	215	98 (45.58)	0.28
	Advanced	82	45 (54.88)	617	323 (52.35)	81	45 (55.56)	0.81
PBA-s Suicidal ideation frequency Present vs. Not Present	Early	389	40 (10.28)	3889	363 (9.33)	625	39 (6.24)	* 0.03
	Moderate	139	13 (9.35)	1265	110 (8.70)	215	21 (9.77)	0.86
	Advanced	82	15 (18.29)	617	100 (16.21)	81	14 (17.28)	0.88
PBA-s Anxiety frequency Present vs. Not Present	Early	389	235 (60.41)	3889	2225 (57.21)	625	316 (50.56)	* <0.01
	Moderate	139	69 (49.64)	1265	691 (54.62)	215	125 (58.14)	0.29
	Advanced	82	48 (58.54)	617	333 (53.97)	81	36 (44.44)	0.17
PBA-s Irritability frequency Present vs. Not Present	Early	389	228 (58.61)	3889	2124 (54.62)	625	303 (48.48)	* <0.01
	Moderate	139	72 (51.80)	1265	690 (54.55)	215	106 (49.30)	0.33
	Advanced	82	49 (59.76)	617	353 (57.21)	81	46 (56.79)	0.90
PBA-s Angry or aggressive behavior frequency Present vs. Not Present	Early	389	129 (33.16)	3889	1091 (28.05)	625	135 (21.60)	* <0.01
	Moderate	139	41 (29.50)	1265	417 (32.96)	215	49 (22.79)	* 0.01
	Advanced	82	29 (35.37)	617	238 (38.57)	81	24 (29.63)	0.27
PBA-s Lack of initiative (apathy) frequency Present vs. Not Present	Early	389	209 (53.73)	3889	1944 (49.99)	625	264 (42.24)	* <0.01
	Moderate	139	88 (63.31)	1265	884 (69.88)	215	147 (68.37)	0.27
	Advanced	82	63 (76.83)	617	469 (76.01)	81	62 (76.54)	0.98
PBA-s Perseverative thinking or behavior frequency Present vs. Not Present	Early	389	128 (32.90)	3889	1493 (38.39)	625	210 (33.60)	* 0.01
	Moderate	139	76 (54.68)	1265	759 (60.00)	215	119 (55.35)	0.25
	Advanced	82	53 (64.63)	617	408 (66.13)	81	50 (61.73)	0.72
PBA-s Obsessive-Compulsive Behaviors frequency Present vs. Not Present	Early	389	100 (25.71)	3889	800 (20.57)	625	96 (15.36)	* <0.01
	Moderate	139	42 (30.22)	1265	353 (27.91)	215	49 (22.79)	0.22
	Advanced	82	33 (40.24)	617	207 (33.55)	81	20 (24.69)	0.11
PBA-s Delusions frequency Present vs. Not Present	Early	389	26 (6.68)	3889	198 (5.09)	625	21 (3.36)	* 0.05
	Moderate	139	13 (9.35)	1265	124 (9.80)	215	12 (5.58)	0.14
	Advanced	82	24 (29.27)	617	120 (19.45)	81	10 (12.35)	* 0.02
PBA-s Hallucinations frequency Present vs. Not Present	Early	389	6 (1.54)	3889	47 (1.21)	625	7 (1.12)	0.82
	Moderate	139	7 (5.04)	1265	36 (2.85)	215	1 (0.47)	* 0.02
	Advanced	82	17 (20.73)	617	105 (17.02)	81	6 (7.41)	* 0.05
PBA-s Disoriented Behavior frequency Present vs. Not Present	Early	389	78 (20.05)	3889	666 (17.13)	625	104 (16.64)	0.31
	Moderate	139	54 (38.85)	1265	534 (42.21)	215	102 (47.44)	0.23
	Advanced	82	49 (59.76)	617	436 (70.66)	81	58 (71.60)	0.12

* Significant at level p < 0.05. PBA-s = Problem Behaviours Assessment – Short, UHDRS = Unified Huntington’s Disease Rating Scale, HADS-SIS = Hospital Anxiety and Depression Scale – Snaith Irritability Scale.

At moderate stages of disease, late onset participants were significantly more likely than young onset participants to have worse HADS-SIS depression (8.00 [4.00–11.00] vs. 5.00 [3.00–8.00], p < 0.001) and HADS-SIS inward irritability (2.50 [1.00–4.00] vs. 1.00 [0.00–3.00], p = 0.006) (Table 3, Appendix A). HADS-SIS depression and anxiety scores are normal 0-7, borderline 8–10, and abnormal 11–21 [20]. HADS-SIS inward irritability scores are normal <4 and outward irritability scores are normal <5 [21].

Behavioral symptom profiles also differed significantly between the typical onset group and the late onset group at early stages of disease. At early stages of disease, typical onset participants were significantly more likely than late onset participants to abuse drugs and alcohol, have worse anxiety, depression, irritability, apathy, executive function, suicidal ideation frequency, angry or aggressive behavior, lack of initiative, and obsessive-compulsive behaviors (Table 3, Appendix A).

At moderate stages of HD, typical onset participants scored significantly worse compared to late onset participants on drug abuse (6.66% vs. 1.87%, p = 0.004) and PBA-s Angry or aggressive behavior frequency (32.96% vs. 22.79%, p = 0.003) (Table 3, Appendix A).

### Disease Progression

With exclusions applied to remove variability in time of follow-up assessments, 1,467 manifest HD participants were included in the sensitivity analysis determining average change in TFC score. The average decline in mean TFC score from baseline to second visit (1.5 to 2.5 years) was significantly faster in the young onset participants (–1.75 points) compared to the typical (–1.23 points, p = 0.0105) or late onset (–0.97 points, p = 0.0017) participants. From baseline to third visit (2.5 to 3.5 years), the young onset participants again declined significantly faster (–2.27 points) when compared to the late onset group (–1.28 points, p = 0.0002), and the typical group declined faster than the late onset group (–1.81 points, p = 0.005) (Figure A).

**Figure A:**
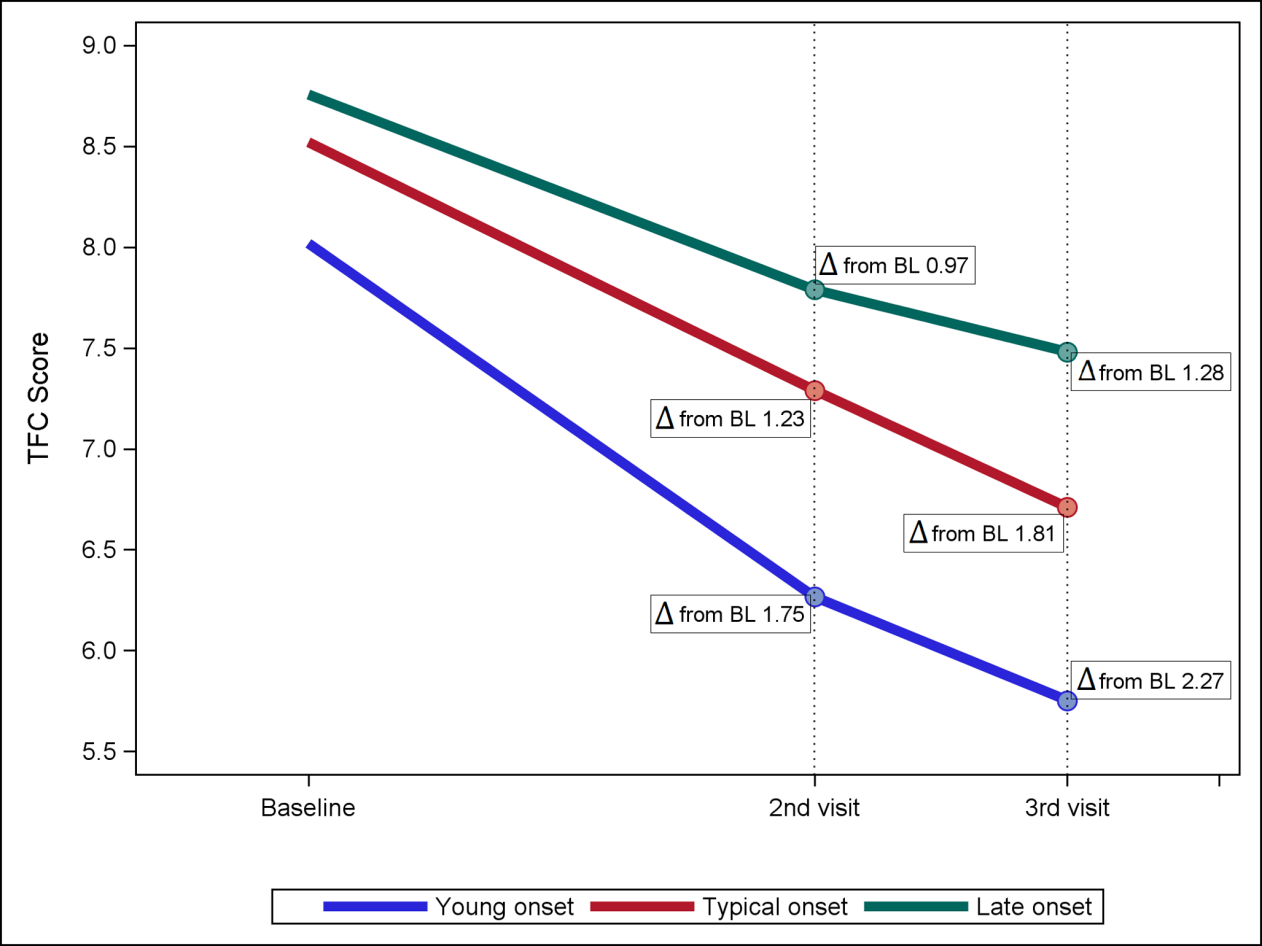
**Average change in TFC score by age-of-onset groups.** Young age of onset group shows markedly increased rate of decline compared to typical and late onset groups. Δ = difference in mean TFC score, BL = baseline.

1,406 manifest HD participants were included in the sensitivity analysis determining average change in UHDRS Functional Assessment Independence Scale. Results reinforced the changes seen in TFC score: the average decline in mean Independence Scale score from baseline to second visit was significantly faster in the young onset participants (–3.53 points) compared to the typical (–2.35 points, p = 0.002) or late onset participants (–2.01 points, p = 0.001). From baseline to third visit, the young onset group again declined significantly faster (–4.62 points) compared to the typical (–3.52 points, p = 0.013) and late onset (–2.92 points, p = 0.001) groups (Figure B).

**Figure B:**
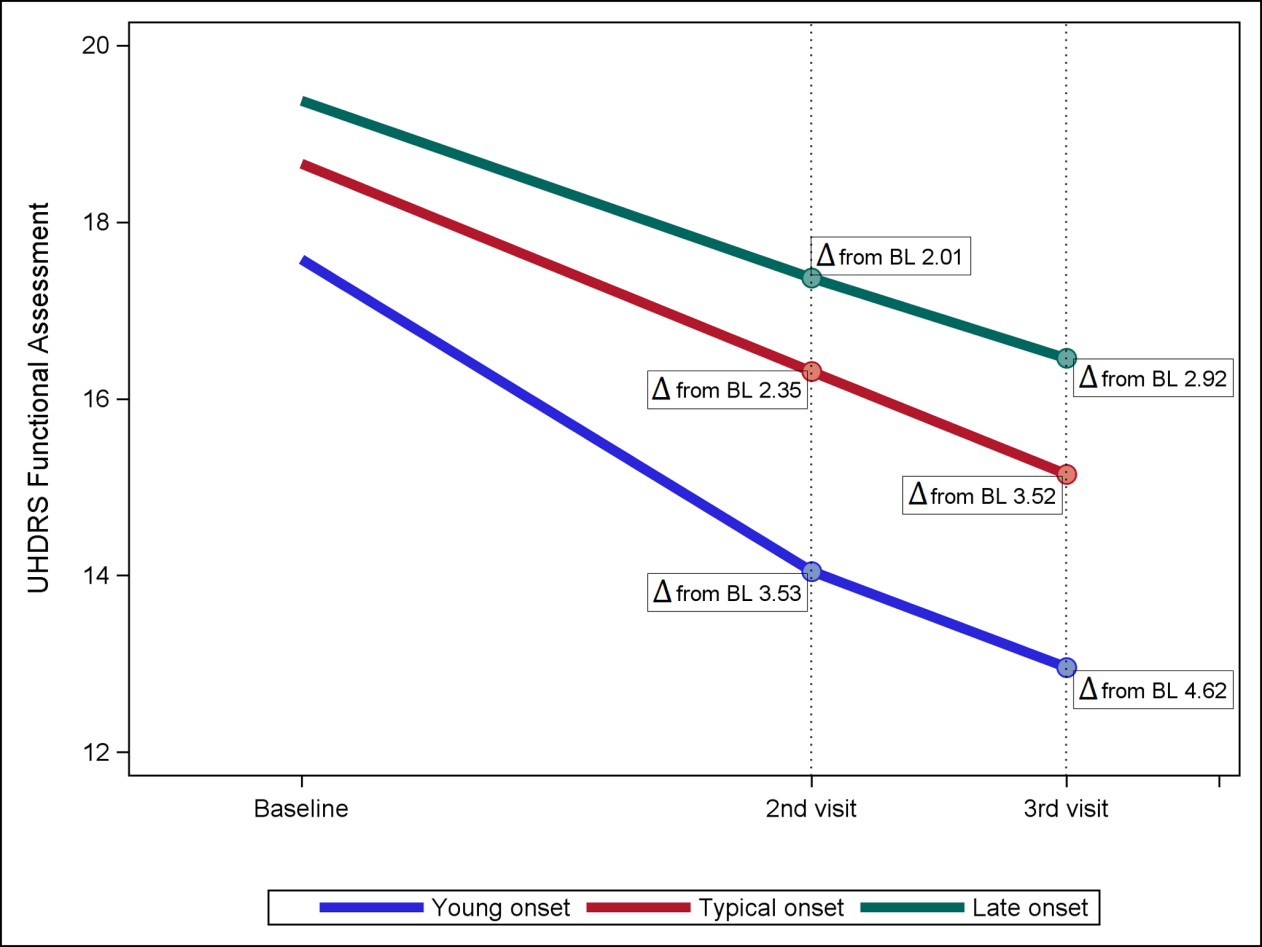
**Average change in UHDRS Functional Assessment Independence Scale.** Young age of onset shows markedly increased rate of decline compared to typical and late onset groups from baseline to second visit and third visit. Δ = difference in mean FA score, BL = baseline.

## Discussion

This analysis corroborates previous findings that symptom burden profile and HD progression differ significantly by age of onset [4]. Knowledge of the expected symptoms profile for different age of onset groups throughout disease progression will help to inform appropriate care of HD patients. Further, a strength of this study is that it clearly defines statistically significant phenotypic differences between the given age groups. Young adult onset (20–29 years) is a novel age group proposed by this study, and our analysis validates the separation of this cohort from the typical onset group based on distinct differences in symptom profile and most clearly in terms of rate of disease progression (Figure A and B). This study distinguishes young adult onset as a clinically unique phenotype, similar to the distinction of juvenile Huntington’s disease, which is defined by features of increased behavioral symptoms and faster progressors in functional decline [10,12,22]. While we recognize these differences likely exist on a continuum rather than stop at our defined age of onset cutoff point, our study shows that younger skewing adult onset participants warrant special considerations in management of their HD.

Regarding behavioral symptoms, at early stages of disease, young onset participants were more likely than late onset participants to suffer from worse behavioral symptoms, including drug and alcohol abuse, anxiety, depression, irritability, aggression, apathy, lack of initiative, obsessive-compulsive behaviors, and delusions. At moderate stages of disease, this preponderance of behavioral symptom burden diminishes, and late onset participants were found to have more depression and inward irritability when compared to the young onset group. At advanced stages of disease, the young onset group was again found to have a greater prevalence of drug abuse, hallucinations, and delusions than the late onset group.

A similar trend was also found in early and moderate stages of disease when comparing the typical onset group to the late onset group. At early stages of disease, the typical onset participants were more likely than late onset participants to abuse drugs and alcohol, have anxiety, depression, irritability, apathy, decreased executive function, increased suicidal ideation, angry or aggressive behavior, lack of initiative, and obsessive-compulsive behaviors. At moderate stages of disease, typical onset participants were more likely than late onset participants to abuse drugs and have angry or aggressive behavior.

These findings support previous suggestions that HD patients with young age of onset have a greater behavioral symptom burden at early disease stage, while late age-of-onset patients have greater motor burden at early stages [4]. Additionally, these findings reinforce our clinical observations, in which younger onset patients are more psychiatrically affected than older onset patients with the same degree of functional impairment. This study builds upon those findings by showing that typical onset participants have a significantly greater burden of behavioral symptoms profiles when compared to late onset participants at both early and moderate stages of disease. Further, although it varies by disease state evaluated, HADS-SIS score has been shown to have a minimal clinically important difference of about 1.5; thus even the small intergroup differences described in this study can affect symptom presentation and management [23].

Additionally, our study makes the important distinction that after adjusting for TFC score, young and typical age of onset participants still had a greater burden of behavioral symptoms compared to the late onset group. Traditionally, expanded CAG allele repeats associated with more aggressive disease have been used to explain the increased burden of behavioral symptoms in people with younger age of onset [1]. However, differences in symptom profiles were observed between the age of onset groups when compared at the same stage of disease severity (Table 3). These findings are corroborated by a previous study, which showed no correlation between CAG repeat length and psychiatric symptoms in HD [24]. Thus, other factors may explain the observed behavioral differences.

Social factors and anxiety about disease progression likely play into the behavioral differences observed. Young people in families with HD endure considerable anxiety and have a lack of support in relation to their HD risk [25]. This burden of anxiety and lack of social support could become magnified once HD manifests. Specifically, lack of social support is associated with decreased quality of life for patients with neurodegenerative diseases [26]. Additionally, given their young age of onset and the genetic nature of HD, these participants are more likely to be caretakers for living family members affected by HD, which has been shown to cause high rates of family dysfunction, psychological stress, and significantly affect quality of life [27,28,29]. Even when controlling for CAG repeat length, HD patients with a family history of HD have been shown to have earlier depression onset and are more likely to have behavioral manifestations as their initial major symptom compared to de novo HD patients [30]. Thus, the young age of onset group may have to deal with the emotional stress of their HD, which is potentially compounded by the emotional stress of caring for ailing HD family members [31].

Besides caring for affected relatives, young age of onset HD patients may also have to manage functional decline with the social expectations of young adult life, such as family planning and employment [31]. Notably, 78.89% of the young onset participants were unemployed, compared to 77.41% in typical onset and 91.40% in late onset (Table 1). While the unemployment rate is not significantly higher in the young onset group, loss of employment at a younger age could be an exacerbating factor for functional decline in the young onset population. There is compelling evidence that the cognitive and functional decline due to HD could cause loss of employment and contribute to exacerbation of behavioral symptoms. A previous study utilizing Enroll-HD found that the functional declines associated with HD contributed to HD patients leaving the workforce earlier [32]. Leaving the labor force has been found to be associated with higher risk of poor physical and mental health in adults [33]. This increased risk for mental illness is likely exacerbated in the young onset group, given they have less opportunity to participate in the workforce compared to late onset participants.

With respect to motor symptoms, this analysis shows that at early disease stages, the late onset group had worse motor function compared to the young onset group. At advanced stages of disease, young onset participants tended to have worse motor function than their late onset counterparts. Therefore, this study indicates that the greater burden of motor symptoms in the late onset group does not persist into more severe disease. At early disease stages, participants with late onset HD potentially have more motor deficits due to natural loss of function from aging. At later stages, HD patients are likely all similarly affected by injury to striatal networks. Further studies are warranted to elucidate why the young onset group had worse motor function with more advanced HD.

Our study found no significant trends regarding differences in cognitive variables between age groups and across stages of disease. This is somewhat surprising, given that age is a risk factor for dementia and cognitive decline [34]. Cognitive decline may be difficult to predict given the number of factors that influence cognitive reserve, such as exercise levels, intelligence level, occupational status, and years of education [34,35,36]. However, the Enroll-HD study participants analyzed in our study were relatively matched in education level and employment status (Table 1); therefore, we can expect all groups would have relatively equal levels of cognitive reserve.

The evidence relating cognitive decline in HD to age of onset prior to this study have been mixed. A previous study found cognitive status was better preserved in younger onset HD patients when compared to later onset patients [37]. Other studies suggested found that CAG repeats, which are correlated with earlier onset, were strongly associated with striatal atrophy and that subcortical atrophy, specifically atrophy of the head of the caudate nucleus, were positively associated with cognitive deficits [1,38,39]. More aggressive disease in the young onset group could potentially be equal to the cognitive deficits caused by aging in the late onset group, thus leading to our result of no significant intergroup differences in cognitive function.

Finally, young age of onset is predictive of a faster functional decline for adults with HD when compared to those with typical and late age of onset. This finding is consistent with the fact that younger age of onset is associated with expanded CAG repeats, which have been associated with faster and more widespread basal ganglia atrophy [1,40,41]. This result directly contradicts the highly cited 1995 study by Feigin et al. which reported that there was no correlation found between functional decline and age of onset in 129 manifest HD patients studied [5]. A 2003 study published by Mahant et al. had results more consistent with ours: in 1,026 patients, the rate of decline in UHDRS total motor score and Independence Scale was significantly faster with a younger age of onset [6]. However, their study included patients with juvenile onset HD and found no association with total functional capacity.

This study makes the important distinction that the association between faster functional decline and younger age of onset persists in the context of adult HD. Additionally, this study was able to show a novel correlation between younger age of onset and a global measure of functional capacity, TFC score. This study shows these robust associations in larger and more diverse HD population followed over the course of 3 years. These findings, paired with the finding that younger age of onset participants had a greater burden of behavioral symptoms, suggest that behavioral symptoms may contribute more significantly to functional decline in this younger population.

Our findings have important implications for the treatment and prognosis of HD. Particularly, patients with a young and typical age of onset should be screened for behavioral symptoms and directed towards resources which will help them more effectively manage these symptoms. This study can help family members and caretakers become more aware that behavioral issues are likely to manifest in patients with earlier HD onset. Given that motor symptoms are worse for young onset patients at later stages of disease, preventative and early interventions with modalities such as physical therapy can be implemented into their care plans [42]. Similar adaptations of our findings can be implemented to tailor treatments and interventions for typical and late onset HD patients.

### Limitations

Since this study was cross-sectional and observational, this precludes us from making causative explanations between the variables studied and limits our conclusions to associations. Although we used rater determination of symptom onset, this estimation of age of onset could still be imprecise and amount to some discrepancy in the actual age of onset of manifest HD and the group assigned in this study. Study participants were categorized based on their TFC score at baseline and the motor, cognitive, and behavioral variables analyzed were determined from baseline visits. This cross-sectional design meant we could not account for interpersonal differences in the participant population. We are limited by the amount of follow-up data currently available in Enroll-HD. Sufficient TFC data points to analyze functional decline were only available up to visit 3, and these participants represented 20.88% of manifest HD patients in the Enroll-HD database. Insufficient powering also limited analyses at advanced stages of disease, since there were less than 1,000 participants at TFC Stages IV and V.

### Future directions

Future longitudinal analyses are necessary to expand on and elucidate the findings of this study. Particularly, a multifactorial cause for the greater burden of behavioral symptoms in younger onset HD patients should be investigated. As the Enroll-HD database continues to grow, future studies may be able to better examine the disease course and phenotype of HD, particularly in its advanced stages.

## Conclusions

Young age of onset is predictive of a faster functional decline for adults with HD when compared to those with typical and late age of onset. HD patients with a young age of onset have a greater burden of behavioral symptoms at early stages of disease, suggesting that behavioral symptoms may contribute more significantly to functional decline in this younger population. Motor deficits are comparatively worse for late onset participants at early stages of HD, and worse at advanced stages for young onset participants.

## Additional File

The additional file for this article can be found as follows:

10.5334/tohm.536.s1 Appendix Table A. Pairwise Comparison after Bonferroni P-Value Correction of Motor, Cognitive, and Behavioral Variables.
