# Supplementary material for: Influence of Age of Onset on Huntington’s Disease Phenotype
Source: Tremor Other Hyperkinet Mov (N Y). 2020 Jul 9;10:21. doi: 10.5334/tohm.536 (PMC7394225; doi:10.5334/tohm.536)
Supplement: Appendix Table A. — Pairwise Comparison after Bonferroni P-Value Correction of Motor, Cognitive, and Behavioral Variables. [file tohm-10-1-536-s1.pdf]

Appendix Table A. Pairwise Comparison after Bonferroni P-Value Correction of Motor, Cognitive, and Behavioral Variables

Variable of interest	TFC stage	Typical VS Young	Late VS Typical	Late VS Young
UHDRS Motor score (TMS)	Early	0.04062	**0.00030**	**0.00007**
UHDRS Motor score (TMS)	Advanced	**0.00257**	**0.00001**	**<0.00001**
Timed Up and Go Total time	Early	**0.00958**	**<0.00001**	**<0.00001**
Timed Up and Go Total time	Moderate	0.50785	**0.00771**	0.02601
Symbol Digit Modality Test Total correct %	Early	0.16589	**0.01149**	0.57903
Verbal Fluency Test Total correct % Category	Early	**0.00319**	0.94131	**0.01278**
Verbal Fluency Test Total correct % Category	Advanced	0.42998	**0.01217**	0.01752
Verbal Fluency Test Total correct % Letter	Moderate	0.47843	**0.00271**	**0.00568**
Stroop Interference Test Total correct %	Moderate	0.47829	**0.01120**	0.02077
Has the participant ever abused drugs	Early	**<0.00001**	**<0.00001**	**<0.00001**
Has the participant ever abused drugs	Moderate	**<0.00001**	**0.00437**	**<0.00001**
Has the participant ever abused drugs	Advanced	**0.00241**	0.09620	**0.00068**
Has the participant had alcohol problems in the past	Early	0.02027	**0.00009**	**<0.00001**
HADS-SIS anxiety subscore	Early	0.15660	**<0.00001**	**<0.00001**
HADS-SIS depression subscore	Early	0.45495	**0.00027**	**0.00097**
HADS-SIS depression subscore	Moderate	**0.00039**	0.24090	**0.00045**
HADS-SIS irritability subscore	Early	**0.00824**	**0.00019**	**<0.00001**
HADS-SIS outward irritability subscore	Early	**0.00018**	**0.00060**	**<0.00001**
HADS-SIS inward irritability subscore	Early	0.63533	**0.00133**	**0.01159**
HADS-SIS inward irritability subscore	Moderate	0.07906	0.03117	**0.00616**
Problem Behaviours Assessment (PBA) Depression	Early	0.01818	**<0.00001**	**<0.00001**
PBA Irritability aggression	Early	0.02614	**0.00003**	**0.00001**

Variable of interest	TFC stage	Typical VS Young	Late VS Typical	Late VS Young
PBA Apathy	Early	0.11377	**0.00006**	**0.00005**
PBA Executive function	Early	0.35571	**0.00078**	0.14677
PBA-s Depressed mood	Early	0.08901	**<0.00001**	**<0.00001**
PBA-s Suicidal ideation	Early	0.54135	**0.01171**	0.01951
PBA-s Anxiety	Early	0.22368	**0.00186**	**0.00220**
PBA-s Irritability	Early	0.13092	**0.00430**	**0.00168**
PBA-s Angry or aggressive behavior	Early	0.03336	**0.00076**	**0.00005**
PBA-s Angry or aggressive behavior	Moderate	0.40777	**0.00298**	0.15708
PBA-s Lack of initiative (apathy)	Early	0.15949	**0.00032**	**0.00036**
PBA-s Perseverative thinking of behavior	Early	0.03347	0.02183	0.81939
PBA-s Obsessive-Compulsive Behaviors	Early	0.01780	**0.00243**	**0.00005**
PBA-s Delusions	Early	0.17881	0.06151	**0.01437**
PBA-s Delusions	Advanced	0.03887	0.12261	**0.00784**
PBA-s Hallucinations	Moderate	0.18681	0.03318	**0.00699**
PBA-s Hallucinations	Advanced	0.40517	0.02617	**0.01456**

Bolded *= significant at level p<0.01667 (Bonferroni Correction)*

PBA-s = Problem Behaviours Assessment – Short, UHDRS = Unified Huntington's Disease Rating Scale, HADS-SIS = Hospital Anxiety and Depression Scale - Snaith Irritability Scale
